# Supplementary material for: Multi‐dataset identification of innovative feature genes and molecular mechanisms in keratoconus
Source: J Cell Mol Med. 2024 Sep 19;28(18):e70079. doi: 10.1111/jcmm.70079 (PMC11412914; doi:10.1111/jcmm.70079)
Supplement: Supplementary file 2 — Material S2. [file JCMM-28-e70079-s002.zip › 7.GSEA analysis/5.RNF217-AS1/KEGG_OXIDATIVE_PHOSPHORYLATION.html]

Details for gene set KEGG\_OXIDATIVE\_PHOSPHORYLATION[GSEA]

|  || Dataset | RNF217-AS1.group.cls#h\_versus\_l |
| Phenotype | group.cls#h\_versus\_l |
| Upregulated in class | l |
| GeneSet | KEGG\_OXIDATIVE\_PHOSPHORYLATION |
| Enrichment Score (ES) | -0.36513186 |
| Normalized Enrichment Score (NES) | -1.590083 |
| Nominal p-value | 0.046464648 |
| FDR q-value | 0.32044238 |
| FWER p-Value | 0.615 |
Table: GSEA Results Summary

  

Fig 1: Enrichment plot: KEGG\_OXIDATIVE\_PHOSPHORYLATION      
 Profile of the Running ES Score & Positions of GeneSet Members on the Rank Ordered List

  

| SYMBOL | TITLE | RANK IN GENE LIST | RANK METRIC SCORE | RUNNING ES | CORE ENRICHMENT || 1 | ATP6V0D2 | na | 2490 | 0.317 | -0.0214 | No |
| 2 | COX7B2 | na | 6236 | 0.176 | -0.1015 | No |
| 3 | ATP6V0D1 | na | 9204 | 0.101 | -0.1711 | No |
| 4 | COX4I2 | na | 11142 | 0.068 | -0.2162 | No |
| 5 | COX7A2L | na | 11823 | 0.058 | -0.2266 | No |
| 6 | COX6B2 | na | 12168 | 0.053 | -0.2281 | No |
| 7 | ATP6V1G3 | na | 12812 | 0.045 | -0.2395 | No |
| 8 | NDUFA10 | na | 14013 | 0.031 | -0.2693 | No |
| 9 | NDUFB5 | na | 14086 | 0.030 | -0.2666 | No |
| 10 | COX7B | na | 14914 | 0.020 | -0.2873 | No |
| 11 | COX6C | na | 15464 | 0.014 | -0.3009 | No |
| 12 | NDUFV1 | na | 15508 | 0.014 | -0.2999 | No |
| 13 | NDUFS6 | na | 15562 | 0.013 | -0.2993 | No |
| 14 | ATP6V0B | na | 15585 | 0.013 | -0.2978 | No |
| 15 | ATP6V1D | na | 15821 | 0.011 | -0.3029 | No |
| 16 | NDUFA1 | na | 15926 | 0.010 | -0.3044 | No |
| 17 | NDUFS4 | na | 16079 | 0.008 | -0.3076 | No |
| 18 | ATP6V0E1 | na | 16390 | 0.005 | -0.3157 | No |
| 19 | NDUFS1 | na | 16420 | 0.004 | -0.3159 | No |
| 20 | NDUFB1 | na | 16574 | 0.003 | -0.3198 | No |
| 21 | PPA2 | na | 16659 | 0.002 | -0.3219 | No |
| 22 | COX6B1 | na | 16711 | 0.002 | -0.3231 | No |
| 23 | COX7C | na | 16867 | -0.000 | -0.3276 | No |
| 24 | NDUFB3 | na | 16984 | -0.001 | -0.3307 | No |
| 25 | COX5B | na | 17009 | -0.002 | -0.3311 | No |
| 26 | SDHD | na | 17106 | -0.003 | -0.3335 | No |
| 27 | UQCRC2 | na | 17167 | -0.003 | -0.3347 | No |
| 28 | COX17 | na | 17279 | -0.004 | -0.3372 | No |
| 29 | UQCR11 | na | 17489 | -0.006 | -0.3423 | No |
| 30 | NDUFAB1 | na | 17544 | -0.007 | -0.3427 | No |
| 31 | NDUFC1 | na | 17617 | -0.007 | -0.3436 | No |
| 32 | NDUFS5 | na | 17643 | -0.008 | -0.3432 | No |
| 33 | COX7A2 | na | 17707 | -0.008 | -0.3437 | No |
| 34 | SDHA | na | 17733 | -0.008 | -0.3431 | No |
| 35 | NDUFA6 | na | 17794 | -0.009 | -0.3434 | No |
| 36 | NDUFB4 | na | 17944 | -0.010 | -0.3461 | No |
| 37 | NDUFS8 | na | 17948 | -0.010 | -0.3445 | No |
| 38 | COX6A1 | na | 18053 | -0.011 | -0.3457 | No |
| 39 | UQCRC1 | na | 18155 | -0.012 | -0.3467 | No |
| 40 | ATP6V1F | na | 18311 | -0.014 | -0.3490 | No |
| 41 | NDUFB9 | na | 18519 | -0.016 | -0.3525 | Yes |
| 42 | UQCRQ | na | 18543 | -0.016 | -0.3506 | Yes |
| 43 | COX8A | na | 18621 | -0.017 | -0.3502 | Yes |
| 44 | NDUFS3 | na | 18677 | -0.017 | -0.3490 | Yes |
| 45 | NDUFV3 | na | 18696 | -0.017 | -0.3468 | Yes |
| 46 | NDUFV2 | na | 18702 | -0.017 | -0.3442 | Yes |
| 47 | ATP6V0C | na | 18707 | -0.017 | -0.3415 | Yes |
| 48 | NDUFB8 | na | 18716 | -0.018 | -0.3389 | Yes |
| 49 | COX6A2 | na | 18729 | -0.018 | -0.3365 | Yes |
| 50 | NDUFA4 | na | 18841 | -0.019 | -0.3367 | Yes |
| 51 | ATP6V1G1 | na | 19140 | -0.021 | -0.3419 | Yes |
| 52 | COX5A | na | 19181 | -0.022 | -0.3396 | Yes |
| 53 | NDUFB7 | na | 19315 | -0.023 | -0.3397 | Yes |
| 54 | ATP6V1C1 | na | 19502 | -0.025 | -0.3410 | Yes |
| 55 | NDUFA9 | na | 19508 | -0.025 | -0.3372 | Yes |
| 56 | NDUFS7 | na | 19536 | -0.026 | -0.3339 | Yes |
| 57 | ATP6V1B2 | na | 19557 | -0.026 | -0.3303 | Yes |
| 58 | NDUFB10 | na | 19641 | -0.027 | -0.3285 | Yes |
| 59 | NDUFA7 | na | 19695 | -0.027 | -0.3257 | Yes |
| 60 | PPA1 | na | 20000 | -0.030 | -0.3297 | Yes |
| 61 | ATP6V1E1 | na | 20165 | -0.032 | -0.3294 | Yes |
| 62 | NDUFA3 | na | 20174 | -0.032 | -0.3246 | Yes |
| 63 | ATP6V1A | na | 20315 | -0.033 | -0.3234 | Yes |
| 64 | UQCRH | na | 20365 | -0.033 | -0.3195 | Yes |
| 65 | COX4I1 | na | 20433 | -0.034 | -0.3160 | Yes |
| 66 | NDUFA4L2 | na | 20479 | -0.035 | -0.3117 | Yes |
| 67 | ATP6AP1 | na | 20488 | -0.035 | -0.3064 | Yes |
| 68 | NDUFA8 | na | 20605 | -0.036 | -0.3040 | Yes |
| 69 | NDUFB2 | na | 20644 | -0.036 | -0.2993 | Yes |
| 70 | COX7A1 | na | 20777 | -0.038 | -0.2972 | Yes |
| 71 | SDHC | na | 20833 | -0.038 | -0.2927 | Yes |
| 72 | NDUFA11 | na | 21093 | -0.041 | -0.2936 | Yes |
| 73 | UQCRFS1 | na | 21182 | -0.042 | -0.2895 | Yes |
| 74 | ATP6V1C2 | na | 21292 | -0.043 | -0.2858 | Yes |
| 75 | NDUFA2 | na | 21309 | -0.043 | -0.2794 | Yes |
| 76 | UQCRB | na | 21369 | -0.044 | -0.2741 | Yes |
| 77 | ATP6V1H | na | 21597 | -0.046 | -0.2733 | Yes |
| 78 | ATP6V0A1 | na | 21733 | -0.048 | -0.2696 | Yes |
| 79 | SDHB | na | 21783 | -0.048 | -0.2633 | Yes |
| 80 | NDUFB6 | na | 21949 | -0.050 | -0.2601 | Yes |
| 81 | UQCR10 | na | 21990 | -0.051 | -0.2532 | Yes |
| 82 | NDUFA5 | na | 22469 | -0.056 | -0.2581 | Yes |
| 83 | COX11 | na | 22929 | -0.061 | -0.2616 | Yes |
| 84 | CYC1 | na | 23861 | -0.073 | -0.2769 | Yes |
| 85 | NDUFC2 | na | 24639 | -0.085 | -0.2858 | Yes |
| 86 | NDUFS2 | na | 25676 | -0.101 | -0.2996 | Yes |
| 87 | LHPP | na | 26001 | -0.107 | -0.2919 | Yes |
| 88 | ATP12A | na | 26563 | -0.117 | -0.2894 | Yes |
| 89 | COX15 | na | 29186 | -0.181 | -0.3363 | Yes |
| 90 | ATP4B | na | 29548 | -0.192 | -0.3160 | Yes |
| 91 | ATP6V1E2 | na | 30210 | -0.213 | -0.3011 | Yes |
| 92 | ATP6V0A4 | na | 30469 | -0.223 | -0.2731 | Yes |
| 93 | COX10 | na | 30828 | -0.235 | -0.2460 | Yes |
| 94 | ATP4A | na | 32269 | -0.307 | -0.2386 | Yes |
| 95 | ATP6V1G2 | na | 32923 | -0.355 | -0.2010 | Yes |
| 96 | ATP6V1B1 | na | 32937 | -0.356 | -0.1447 | Yes |
| 97 | ATP6V0E2 | na | 33133 | -0.370 | -0.0913 | Yes |
| 98 | COX8C | na | 34671 | -0.862 | 0.0016 | Yes |
Table: GSEA details [plain text format]

  

Fig 2: KEGG\_OXIDATIVE\_PHOSPHORYLATION      
 Blue-Pink O' Gram in the Space of the Analyzed GeneSet

  

Fig 3: KEGG\_OXIDATIVE\_PHOSPHORYLATION: Random ES distribution      
 Gene set null distribution of ES for **KEGG\_OXIDATIVE\_PHOSPHORYLATION**
